# 2412: Predicting response to hemodynamic interventions in the ICU using recurrent neural networks

**DOI:** 10.1017/cts.2017.73

**Published:** 2018-05-10

**Authors:** Julian Genkins, Thomas A. Lasko

## Abstract

OBJECTIVES/SPECIFIC AIMS: Our goal is to explore the value of learning algorithms to improve both the efficiency and accuracy of a clinician undertaking the cognitive task of selecting the best resuscitative intervention for a hemodynamically unstable patient in the ICU. Machine learning is an ideal discipline to solve this problem. The ICU is a data rich environment, however there is significant uncertainty regarding the interdependency of this data. Experts consistently struggle to develop deterministic models of the underlying forces driving hemodynamic perturbations and intervention responsiveness. Machine learning, especially deep learning, assumes no correlation between inputs. Deep architectures disentangle these high-level relationships through exposure to abundant, diverse data sets such as those used in this project, obviating the need to manually explore confounding interactions. METHODS/STUDY POPULATION: We are using the “Medical Information Mart for Intensive Care” (MIMIC-III) database for this project. MIMIC-III is a large, single-center database comprising information relating to patients admitted to critical care units at Beth Israel Deaconess Medical Center, a large tertiary care hospital, from 2001 to 2012. It contains data associated with 38,597 distinct adult patients and 53,423 distinct hospital admissions for those patients, with a mean of 4579 charted observations and 380 laboratory measurements available for each hospital admission. Classes of data in the MIMIC-III are varied and include billing, intervention, laboratory, medication, and physiologic data among others. In addition to training an RNN in the task of predicting hemodynamic states, we will also attempt to train 2 additional models on the same data—a multidimensional linear regression and a nonsequence-oriented deep neural network. For each of these models we will measure accuracy using root mean squared error (RMSE) and mean absolute error (MAE) to provide scale-dependent measurements of accuracy. RESULTS/ANTICIPATED RESULTS: Our results will be reported in 2 primary categories: numerical accuracy of the RNN model and applicability, utility, and accuracy in a live clinical setting. The use of RNNs in biomedical informatics, and in general, is a relatively new phenomenon. This means that the body of literature which could provide a basis for our expected results is limited. Because of this we have chosen staged goals in assessing our model. First, we hope to achieve a model that reliably predicts the direction of response. Being able to answer only the question of how a patient will respond—will they move toward or away from our therapeutic goal—is as good as existing prediction methods. It is well established in the literature that, by almost any metric, ~50% of hemodynamically unstable patients respond to a fluid challenge. If we are within 10% of this average (40%–60% respond), then we can be confident in the accuracy of our model in predicting direction. Upon achieving this, we will then measure accurate prediction of response magnitude. To this affect, we hope to achieve an RMSE <10% between our test data and corresponding predicted output before proceeding further. In addition to numeric accuracy, we acknowledge that a plan for practical, clinical validation is needed before utilizing this tool in a clinical environment. Such validation will require 3 separate components. First, numeric accuracy will need to be determined again as compared with prospective data on actual patients in the ICU. This step is critical to prove that no information leakage from target data back to input data occurred during training. Second, there must be a comparison to existing prediction methods, such as the passive leg raise in combination with measurement of cardiac output to predict volume responsiveness. Finally, we must measure the cost to the clinician of implementing our model in an ICU, specifically how it impacts their time to accomplish the task of selecting an intervention for the hemodynamically unstable patient. However, these tasks are beyond the scope of this project and will be left for later investigations. DISCUSSION/SIGNIFICANCE OF IMPACT: If we are successful, this study will provide the first step toward a data-driven model for predicting patient responsiveness to a given hemodynamic intervention or collection of interventions. As compared with current best practice maneuvers, this model will not require manipulation of the patient, have less rigid criteria for reliable interpretation, and not require as specific of a technical skillset to interpret. Furthermore, it will include many common categories of resuscitative therapies (eg, vasopressors, inotropes, fluids) and will allow effects of a combination of interventions to be predicted while making no assumptions of interdependence between said interventions. This study will also contribute a novel process of sequence prediction using RNNs by incorporating an element of context on top of the sequential data in every training step. An RNN learning the sequence of hemodynamic data comprising a patient’s hemodynamic state would, alone, fit into the realm of sequence prediction. Our innovation is the addition of treatment information with each temporal division of the hemodynamic data. The result is an RNN that combines the task of sequence prediction with sequence translation, the 2 major use cases for RNN learning algorithms.

